# Deposition of HfO_2_ by Remote Plasma ALD for High-Aspect-Ratio Trench Capacitors in DRAM

**DOI:** 10.3390/nano15110783

**Published:** 2025-05-23

**Authors:** Jiwon Kim, Inkook Hwang, Byungwook Kim, Wookyung Lee, Juha Song, Yeonwoong Jung, Changbun Yoon

**Affiliations:** 1 Department of Advanced Materials Engineering, Tech University of Korea, Siheung-si 15073, Gyeonggi-do, Republic of Korea; jw2001kim@tukorea.ac.kr (J.K.); quddnr000@tukorea.ac.kr (B.K.); ds090390@tukorea.ac.kr (W.L.); 2 School of Chemistry, Chemical Engineering and Biotechnology, Nanyang Technological University, Singapore 637459, Singapore; 3 Department of Materials Science and Engineering, University of Central Florida, Orlando, FL 32816, USA

**Keywords:** remote plasma ALD, thermal ALD, plasma damage, trench structure, atomic layer deposition, leakage current, capacitor, step coverage

## Abstract

Dynamic random-access memory (DRAM) is a vital component in modern computing systems. Enhancing memory performance requires maximizing capacitor capacitance within DRAM cells, which is achieved using high-k dielectric materials deposited as thin, uniform films via atomic layer deposition (ALD). Precise film deposition that minimizes electronic defects caused by charged vacancies is essential for reducing leakage current and ensuring high dielectric strength. In this study, we fabricated metal–insulator–metal (MIM) capacitors in high-aspect-ratio trench structures using remote plasma ALD (RP-ALD) and direct plasma ALD (DP-ALD). The trenches, etched into silicon, featured a 7:1 aspect ratio, 76 nm pitch, and 38 nm critical dimension. We evaluated the electrical characteristics of HfO_2_-based capacitors with TiN top and bottom electrodes, focusing on leakage current density and equivalent oxide thickness. Capacitance–voltage analysis and X-ray photoelectron spectroscopy (XPS) revealed that RP-ALD effectively suppressed plasma-induced damage, reducing defect density and leakage current. While DP-ALD offered excellent film properties, it suffered from degraded lateral uniformity due to direct plasma exposure. Given its superior lateral uniformity, lower leakage, and defect suppression, RP-ALD shows strong potential for improving DRAM capacitor performance and serves as a promising alternative to the currently adopted thermal ALD process.

## 1. Introduction

Dynamic random-access memory (DRAM) is a widely used semiconductor device that serves as the main memory in computers. With the rapid increase in mobile device usage and exponential improvements in CPU performance, DRAM has continuously evolved to meet higher performance demands [1,2,3]. Each DRAM cell, the smallest memory unit, consists of a transistor acting as a switch and a capacitor that stores electric charge. Unlike flash memory, which uses a floating gate to store charges beneath the gate electrode and retain data without a power supply, DRAM relies on separately fabricated capacitors to store charges [4,5,6,7]. As DRAM loses stored information due to charge leakage when reading data from the capacitor, a refresh operation is required to periodically replenish the charge [8,9].

To increase chip yield per wafer and enhance the speed of semiconductor devices, DRAM must undergo continuous process miniaturization, and extending the refresh cycle is essential for improving performance. As a result, new materials and advanced process technologies are being actively developed to maintain high dielectric capacitance [10,11,12]. A central approach to DRAM miniaturization is increasing the dielectric capacitance of capacitors, which is achieved by fabricating high-aspect-ratio trench structures and depositing dielectric films uniformly. In capacitor fabrication, trenches with aspect ratios exceeding 100:1 are formed and arranged laterally to increase integration density [13,14,15]. However, this lateral configuration introduces thickness non-uniformity perpendicular to the deposition direction. To ensure reliable DRAM performance, uniformity of less than 5% between the top and bottom of the trench is required, significantly complicating the process [16].

To maintain sufficient dielectric thickness while achieving an equivalent oxide thickness (T_ox_) below 1 nm and minimizing leakage current, various high-k materials are being explored [17,18,19]. Uniform deposition processes that ensure the high quality of these high-k materials are becoming increasingly important. Currently, ZrO_2_/Al_2_O_3_/ZrO_2_ (ZAZ) composite films are widely used as dielectric materials in DRAM capacitors, but miniaturization imposes physical constraints that ultimately reduce their charge storage capability [20,21,22]. Achieving sufficient capacitance and suppressing charge loss requires a combination of high-k materials and low leakage current density (J). In this regard, materials such as HfO_2_, ZrO_2_, TiO_2_, Al-doped TiO_2_, and SrTiO_3_ have been extensively studied as potential alternatives to the current ZAZ dielectric films. While high-k materials offer improved electrical performance, such as reduced leakage current at low effective oxide thickness (t_ox_), their low band offset with metal electrodes—due to small bandgaps—often necessitates relatively great physical thickness. Additionally, both the choice of deposited material and the deposition process are critical to achieving high capacitance and low leakage current [23,24].

Historically, planar semiconductor devices were able to achieve high-quality thin films using chemical vapor deposition [25,26]. However, as miniaturization has progressed toward three-dimensional structures, such as FinFETs, gate-all-around structures, and trenches, atomic layer deposition (ALD), offering near-perfect step coverage, has become the standard [27,28]. Currently, ALD is widely adopted in semiconductor deposition processes as the optimal technique for achieving uniform thin films on memory devices with trench structures and nanometer-scale thicknesses. ALD operates by depositing one atomic layer at a time, followed by purging and reaction steps, enabling excellent step coverage and uniformity, though its inherently slow deposition rate remains a persistent challenge [29,30].

ALD processes are categorized into thermal ALD and plasma-enhanced (PE) ALD based on how reactive gases are activated. PE-ALD is favored in many semiconductor applications due to its lower process temperature, denser films, and higher deposition rate. Nonetheless, for DRAM capacitors, which use laterally aligned trenches, thermal ALD remains the preferred method despite its lower deposition rate. PE-ALD can be further divided into direct plasma (DP) ALD and remote plasma (RP) ALD. In DP-ALD, plasma is generated directly inside the reaction chamber, energizing gases in situ. While DP-ALD offers high deposition rates and excellent film quality, it can lead to ion bombardment of the substrate or film surface, resulting in interface damage and degradation of film properties [31,32,33]. As DRAM structures evolve into horizontally stacked trench arrays with hundreds of layers, the strong directionality of radicals in DP-ALD, which are guided by the electric field, introduces challenges for uniform film formation. As a result, thermal ALD remains the dominant method for capacitor fabrication. However, for future extreme miniaturization and higher productivity, the implementation of PE-ALD is becoming essential. RP-ALD, which isolates the plasma generation from the process chamber and delivers only activated radicals, has emerged as a promising alternative for mitigating plasma-induced damage and electric-field directionality issues observed in DP-ALD. Nevertheless, due to the limited lifetime of radicals in RP-ALD, the optimization of equipment design and process parameters is necessary, and further research is required to obtain films with the desired properties [34,35].

Despite the widespread use of ALD techniques in DRAM fabrication, there remains a lack of direct comparative analysis between DP-ALD and RP-ALD, specifically for high-aspect-ratio trench structures. Therefore, this study investigates the differences between these plasma-based ALD techniques for trench structures. Hafnium oxide, a representative high-k material, was deposited using both DP-ALD and RP-ALD to examine the impact of plasma on deposition characteristics. Trenches with a 76 nm pitch, 38 nm critical dimension (CD), and an aspect ratio of 7:1 were fabricated, and the resulting deposition profiles were analyzed using scanning electron microscopy (SEM) [36,37,38]. To assess film uniformity, depositions were also performed on 6-inch wafers and evaluated using ellipsometry. Additionally, plasma-induced defects such as oxygen vacancies were investigated. Thin films obtained from the RP-ALD and DP-ALD processes were characterized via X-ray photoelectron spectroscopy (XPS), and their electrical properties, such as dielectric constant and leakage current, were evaluated. Based on leakage current analysis, RP-ALD demonstrated superior performance compared to DP-ALD. Step coverage was also found to be more favorable in RP-ALD than in DP-ALD. However, DP-ALD yielded higher dielectric constant values than RP-ALD, which can be attributed to the presence of oxygen vacancies in the HfO_2_ films. The excellent lateral uniformity and superior thin-film properties observed in RP-ALD suggest that it may serve as a viable replacement for thermal ALD in DRAM capacitor fabrication, potentially enhancing device performance [39,40,41,42,43,44].

## 2. Materials and Methods

### 2.1. Deposition of HfO_2_ Thin Films Using RP-ALD and DP-ALD

**Figure 1:**
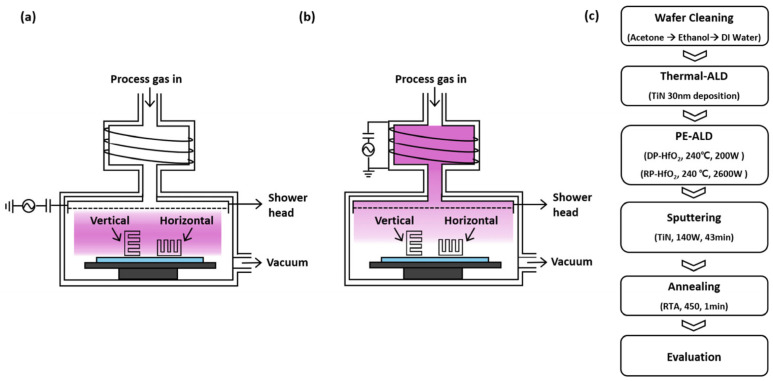
Deposition processes and conditions for laterally and vertically aligned trench-structured substrates simulating DRAM capacitors. (**a**) DP-ALD process. (**b**) RP-ALD process. (**c**) Process flow for HfO*_2_* deposition using DP-ALD and RP-ALD.

Figure 1 shows the silicon trench structure and the process conditions used for the deposition tests. In Figure 1a, a silicon substrate with trenches having an aspect ratio of 7:1, a pitch of 76 nm, and a CD of 38 nm was fabricated and used to evaluate both lateral and vertical deposition using DP-ALD. Figure 1b shows a corresponding setup for deposition using RP-ALD on a similarly structured substrate. These trench structures were fabricated using a test pattern wafer (SK Hynix Inc., Icheon-si, Republic of Korea). Film uniformity was evaluated using an ellipsometer (Elli-SE-U, Ellipso Technology, Daejeon, Republic of Korea) after deposition via both the DP and RP methods.

Figure 1c outlines the detailed process flows for the DP and RP deposition methods. In DP-ALD, plasma was generated directly inside the PE-ALD chamber, where reactive gases such as O_2_ were introduced near the substrate, and a plasma power of 200 W was applied. By contrast, RP-ALD used a remote plasma source (En2ra-RPS, EN2CORE Technology, Daejeon, Republic of Korea), where only radicals activated in a remote plasma zone were introduced into the chamber. To generate a sufficient radical density, approximately 2600 W of remote plasma power was used, and the radicals were delivered to the chamber using Ar as the carrier gas. For the deposition of the high-k dielectric layer, tetrakis(ethylmethylamido)hafnium (TEMA-Hf, iChems, Gyeonggi-do, Republic of Korea) was used as the precursor for HfO_2_ deposition. For electrical property evaluation, a 50 nm thick TiN bottom electrode was first deposited on the trench-structured substrate. HfO_2_ films were then deposited, followed by the formation of top TiN electrodes (200 μm in diameter, 50 nm in thickness) via a lift-off process. The TiN top electrodes were deposited by RF magnetron sputtering at room temperature for 43 min. To minimize defect density within the dielectric films, rapid thermal annealing was performed at 450 °C for 1 min in a nitrogen ambient atmosphere.

### 2.2. Evaluation of the Properties of Trenches Deposited by DP and RP-ALD

The HfO_2_ film thickness was measured using spectroscopic ellipsometry. To assess the uniformity of the DP and RP deposition processes, films were deposited on a 6-inch wafer, and thickness distributions were measured across the substrate, including along the X- and Y-axes, to further confirm the uniformity. The MIM capacitor thin film structure was analyzed using SEM (Nova NanoSEM 450, FEI, Hillsboro, OR, USA) at an accelerating voltage of 10 kV. Film composition and defects in the HfO_2_ films deposited by the DP and RP methods were analyzed and compared using XPS (NEXSA, Thermo Fisher Scientific, Seoul, Republic of Korea). XPS data were collected using an Escalab 250 photoelectron spectrometer (ThermoFisher Scientific, Waltham, MA, USA) using 300 W Al Kα irradiation. Furthermore, the dielectric capacitance and leakage current of the MIM capacitor were measured using a semiconductor characterization system (4200A-SCS, Keithley, Cleveland, OH, USA) connected to a microprobe station (APX-6B, WIT Co., Suwon, Republic of Korea). C–V measurements were conducted at 1 MHz.

## 3. Results

### 3.1. Characteristics of HfO_2_ Thin Films Deposited by DP-ALD and RP-ALD

**Figure 2:**
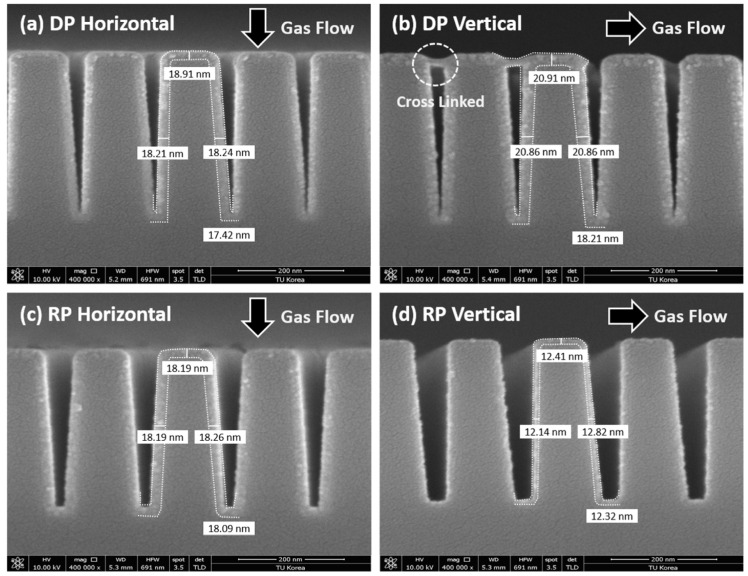
Cross-sectional images of trenches deposited by DP-ALD: (**a**) horizontal deposition; (**b**) vertical deposition. Cross-sectional images of trenches deposited by RP-ALD: (**c**) horizontal deposition; (**d**) vertical deposition.

Figure 2 illustrates the application of DP-ALD and RP-ALD processes for the deposition of hafnium oxide (HfO_2_) thin films on trench-structured substrates. The trenches had a depth of 600 nm, a pattern pitch of 76 nm, and a CD of approximately 38 nm, etched in a bar-type pattern. As shown in Figure 1, the patterns were arranged in both vertical and horizontal orientations, and the ALD processes were conducted using different types of plasma.

The deposition results using DP-ALD and RP-ALD are shown in Figure 2a,b and Figure 2c,d, respectively. For DP-ALD, horizontal deposition (Figure 2a) exhibited high step coverage owing to the perpendicular alignment between the deposition direction and the substrate orientation. By contrast, vertical deposition (Figure 2b) showed lower step coverage because the deposition direction was parallel to the trench orientation, limiting precursor penetration into the trench. In horizontal deposition, the upper and lower film thicknesses were approximately 18.9 and 17.4 nm, respectively, resulting in a step coverage of 0.92. For vertical deposition, the upper thickness was 20.9 nm and the lower was 18.2 nm, yielding a reduced step coverage of approximately 0.87. By contrast, RP-ALD achieved uniform HfO_2_ thin film deposition within the trench regardless of the substrate orientation. In horizontal deposition (Figure 2c), the airflow was perpendicular to the trench, allowing for uniform coating. Even in vertical deposition (Figure 2d), where the deposition direction was parallel to the trench, RP-ALD maintained conformal coverage deep into the trench, unlike DP-ALD.

**Figure 3:**
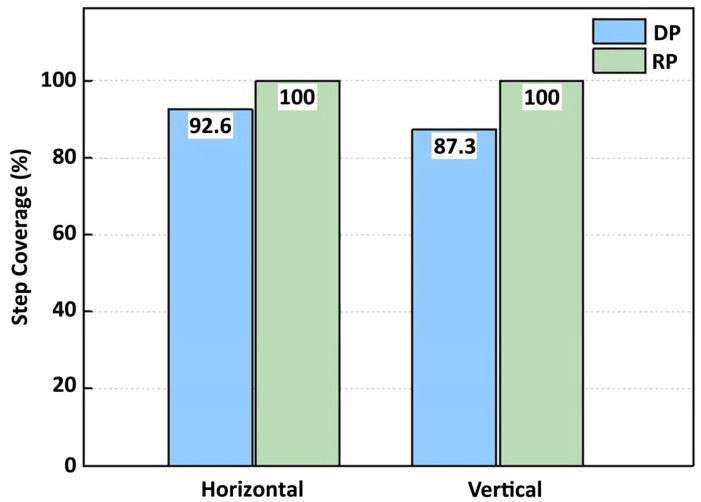
Step coverage results for thin films deposited by DP-ALD and RP-ALD depending on the substrate orientation in the trench structure.

Figure 3 summarizes the step coverage of the ALD processes based on the type of plasma used. For DP-ALD, the step coverage was 92.6% for horizontal deposition and 87.3% for vertical deposition. By contrast, RP-ALD achieved nearly 100% step coverage in both orientations, demonstrating superior deposition uniformity compared to DP-ALD. Regarding deposition rate, DP-ALD exhibited higher productivity, with rates of 0.97 Å/cycle and 1.12 Å/cycle for horizontal and vertical deposition, respectively. In comparison, RP-ALD showed relatively low deposition rates of 0.72 Å/cycle for horizontal deposition and 0.55 Å/cycle for vertical deposition. This difference can be attributed to the higher plasma energy delivered to the substrate in the DP-ALD process, which enhances deposition efficiency. Therefore, while DP-ALD is advantageous in terms of productivity, RP-ALD provides better performance in deposition uniformity.

**Figure 4:**
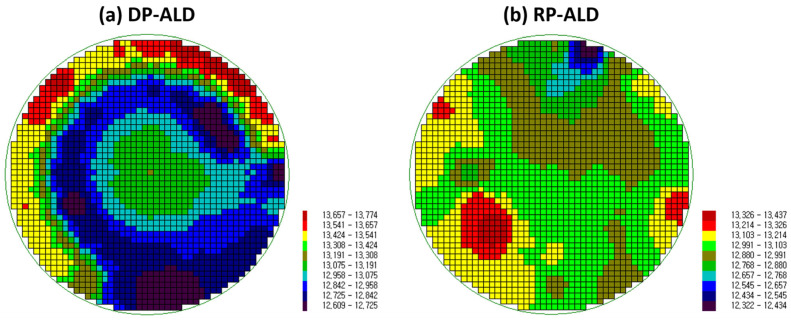
Thickness uniformity measurement results for thin films on a 6-inch (152 mm) wafer based on the deposition type: (**a**) DP-ALD, (**b**) RP-ALD.

Figure 4 presents the thickness distribution of HfO_2_ thin films deposited on 6-inch (152 mm) wafers using DP-ALD and RP-ALD. In DP-ALD, a capacitively coupled plasma system based on RF power is used, resulting in noticeable film thickness non-uniformities, particularly in the central region of the wafer, owing to plasma influence. This variation appears as blue regions, indicating thinner films where plasma effects are stronger, and red regions, indicating thicker films where plasma influence is weaker, producing a distinct thickness gradient across the wafer. By contrast, RP-ALD utilizes an inductively coupled plasma system, where plasma is generated externally, and Ar^+^ ion emission is effectively blocked. Only reactive radicals are introduced into the chamber via the carrier gas, Ar. This configuration prevents ion-induced damage, such as that caused by Ar^+^ ions, thereby enabling uniform thin film deposition. The thickness variations observed in RP-ALD can be attributed to airflow distribution within the chamber rather than plasma-related effects.

**Figure 5:**
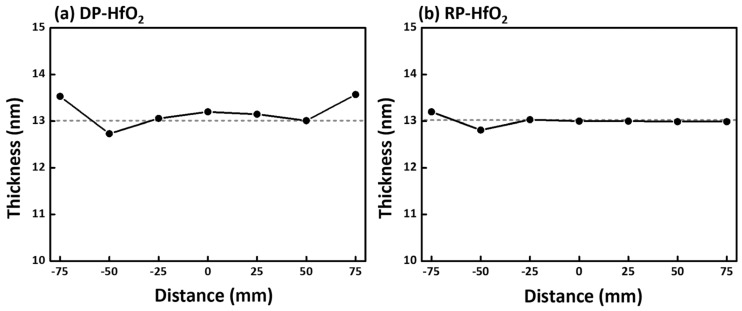
Thickness distribution data for thin films deposited on a wafer: (**a**) DP-HfO_2_ and (**b**) RP-HfO_2_.

Figure 5 shows the thickness distribution of the HfO_2_ thin films, ranging from 12 to 13 nm depending on the type of plasma used. Non-uniformity, defined as (Maximum − Minimum)/(2 × Average), was approximately 5% for DP and 3% for RP. As shown in Figure 5a, the DP case exhibits a symmetric, bowl-shaped thickness profile centered on the wafer, which can be attributed to the influence of plasma. By contrast, the RP case in Figure 5b shows localized regions of thicker and thinner film, which appear to correlate with both the precursor injection position and the direction of gas exhaust. Thickness uniformity is a critical factor in plasma-enhanced ALD processes, as it directly impacts semiconductor device performance and manufacturing yield.

**Figure 6:**
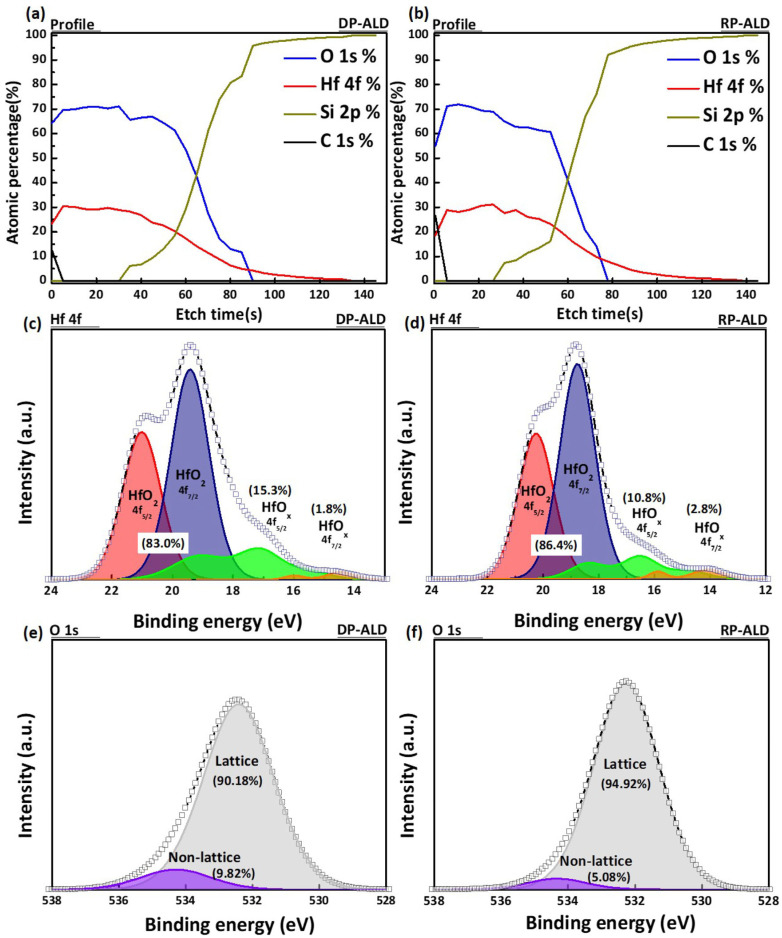
XPS measurement data for HfO_2_ thin films fabricated via DP-ALD and RP-ALD: (**a**,**b**) depth profiling; (**c**,**d**) Hf 4f; (**e**,**f**) O 1s patterns.

To investigate the defect density of the thin films based on plasma type, HfO_2_ films deposited by DP-ALD and RP-ALD were analyzed using X-ray photoelectron spectroscopy (XPS). Figure 6a,b show the XPS depth profile results, which assess elemental composition as a function of etching time. In both cases, the presence of C 1s within the films was negligible, suggesting that the detected surface carbon contamination originated from exposure during the FIB pre-treatment process, with minimal carbon content inside the films. This indicates that the deposition was conducted under optimal conditions. Figure 6c,d show the XPS spectra of Hf 4f for the films deposited by DP-ALD and RP-ALD, respectively. The intensity ratio between Hf 4f52 and Hf 4f72 was fixed at 3:4, and the peaks were deconvoluted into non-lattice Hf^x+^ and metallic Hf^0^ components. The proportion of non-lattice Hf was calculated to be 17.0% for DP-HfO_2_ and 13.6% for RP-HfO_2_, indicating a higher concentration of plasma-induced non-lattice Hf in the DP-HfO_2_ film. Similarly, Figure 6e,f present the deconvoluted O 1s spectra, separating lattice and non-lattice components corresponding to oxygen-related defects. The proportion of non-lattice oxygen was determined to be 9.82% for DP-HfO_2_ and 5.08% for RP-HfO_2_, confirming that the DP-HfO_2_ film contains a higher density of defects compared to the RP-HfO_2_ film.

### 3.2. MIM Device Electrical Characteristics

**Figure 7:**
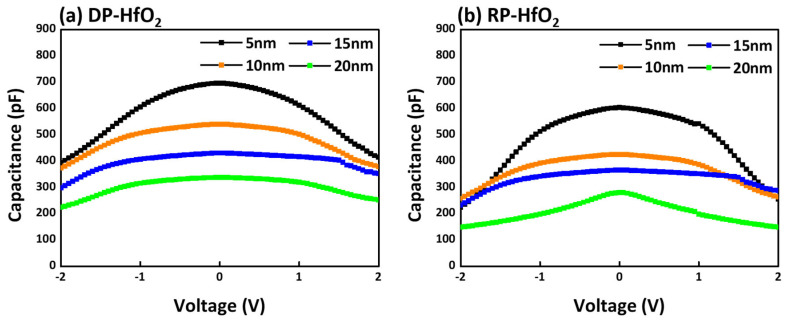
Capacitance measurement results for 5–20 nm HfO_2_ samples fabricated via DP-ALD and RP-ALD, using electrodes with a diameter of 200 μm.

Figure 7 shows a comparison of the capacitance characteristics of DP-HfO_2_ and RP-HfO_2_ thin films with thicknesses ranging from 5 to 20 nm, measured at a frequency of 1 MHz. For dielectric films fabricated by both processes, C–V curves were obtained over a voltage sweep range of −2 V to +2 V, and all films exhibited typical and stable capacitance behavior throughout the measurement range. For 5 nm thick HfO_2_ films, the measured capacitance values were approximately 670 pF for DP-HfO_2_ and 620 pF for RP-HfO_2_. These results indicate that, under identical thickness and electrode area conditions, DP-HfO_2_ exhibits approximately 7.5% higher capacitance compared to RP-HfO_2_. Dielectric films exposed to plasma typically experience degradation in electrical properties owing to interfacial damage and increased leakage current. However, under certain conditions, plasma-induced damage has also been reported to enhance dielectric capacitance. In the case of HfO_2_ thin films deposited by plasma-assisted ALD, this anomalous behavior can be primarily attributed to the formation of oxygen vacancy (V_o_) defects induced by ion bombardment from the plasma. Exposure to plasma can cause the dissociation of oxygen atoms at the HfO_2_ film surface or the metal–dielectric interface, increasing the concentration of oxygen vacancies. These defects act as donor-like charge states and generate localized electric dipoles within the dielectric layer. When subjected to an external electric field, these dipoles can reorient, enhancing dielectric polarization and increasing the overall dielectric permittivity. This behavior can be explained by the “defect–polarization model”, which suggests that changes in the dipole moment of oxygen vacancies contribute to an increase in the real component of permittivity, particularly under low-frequency electric fields. While a moderate level of plasma-induced defects may benefit dielectric capacitance enhancement, excessive defect generation can result in increased leakage current and compromised long-term reliability. Therefore, precise control over the concentration and spatial distribution of oxygen-related defects is essential for optimizing DRAM capacitor performance [45,46].

**Figure 8:**
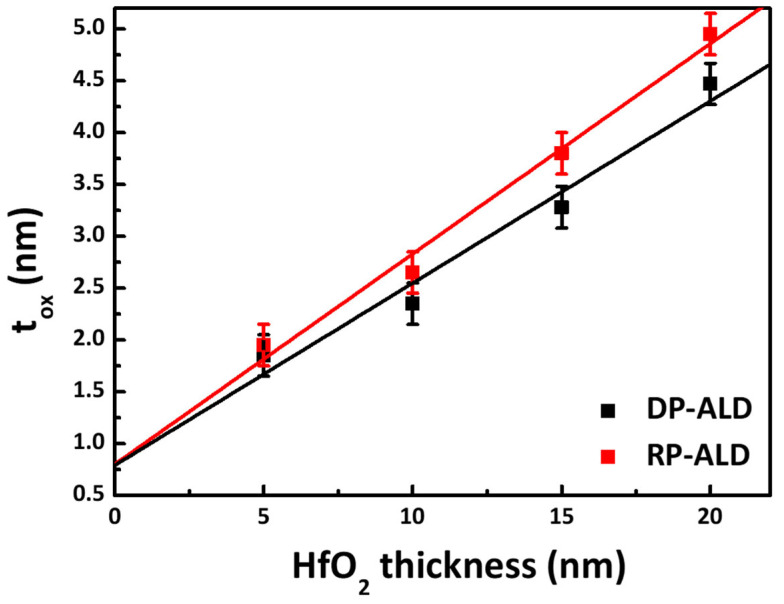
EOT measurement results for HfO_2_ thin films with varying thicknesses fabricated via DP-ALD and RP-ALD.

Figure 8 illustrates the relationship between the physical thickness (t_phy_) and the equivalent oxide thickness (EOT) of HfO_2_ thin films in metal–insulator–metal (MIM) structures as a function of the deposition method. The two deposition methods compared are DP-ALD and RP-ALD. EOT values were calculated using Equation (1), with the physical thickness of the HfO_2_ films ranging from 5 to 20 nm.

(1) $$EOT=\frac{\varepsilon_{{SiO}_{2}\;}\times {\;t}_{high-k}}{\varepsilon_{high-k}}$$

where ε_high-K_ is the dielectric constant of HfO_2_, and t_high-K_ is the thickness of the HfO_2_ layer.

A linear relationship was observed between EOT and physical thickness (t_phy_). For the HfO_2_ films deposited via DP-ALD, linear regression yielded the following equation:

EOT = 0.175 × t_phy_ + 0.79 (2)

The slope corresponds to a dielectric constant of approximately 22.2, which reflects the intrinsic permittivity of the HfO_2_ films, excluding the influence of any interfacial low-k layers. Similarly, for RP-ALD, the relationship is as follows:

EOT = 0.20 × t_phy_ + 0.80 (3)

where the slope corresponds to a dielectric constant of approximately 19.2. The y-intercepts (0.79 nm for DP and 0.80 nm for RP) can be attributed to interfacial oxidation effects, such as the formation of a fixed interfacial layer composed of SiO_2_. The variation in extracted dielectric constants may result from differences in film density, crystallinity, or impurity levels introduced by each deposition method. These results highlight the critical role of deposition parameters in determining the electrical performance of high-k dielectric films [9,47,48].

**Figure 9:**
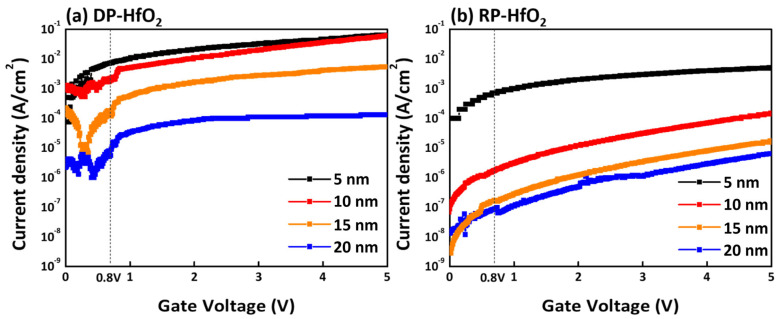
Current density results for 5–20 nm HfO_2_ thin films fabricated via (**a**) DP-ALD and (**b**) RP-ALD.

Figure 9 presents the leakage current characteristics of HfO_2_ thin films deposited using the DP method (Figure 9a) and the RP method (Figure 9b). Leakage current measurements were conducted by applying voltage from 0 to 5 V in 0.05 V increments. As observed, HfO_2_ thin films fabricated via DP-ALD exhibited a relatively high leakage current, which can be attributed to the presence of a large number of defects within the film. By contrast, the films deposited by RP-ALD showed significantly reduced leakage current. This difference can be primarily attributed to the distinct plasma generation mechanisms of each process. In the DP-ALD method, plasma discharge occurs directly within the deposition chamber, exposing the film surface to direct plasma bombardment. However, in the RP-ALD method, plasma is generated in a remote chamber, thereby preventing direct plasma exposure to the film surface. As a result, surface damage and defect formation are effectively suppressed, leading to improved electrical performance over the entire voltage range. Specifically, at 0.8 V and a thickness of 15 nm, the DP-HfO_2_ film exhibited a leakage current density of approximately 10^−4^ A/cm^2^, whereas the RP-HfO_2_ film showed a significantly lower leakage current density of approximately 10^−7^ A/cm^2^, demonstrating three orders of magnitude improvement in leakage performance.

**Figure 10:**
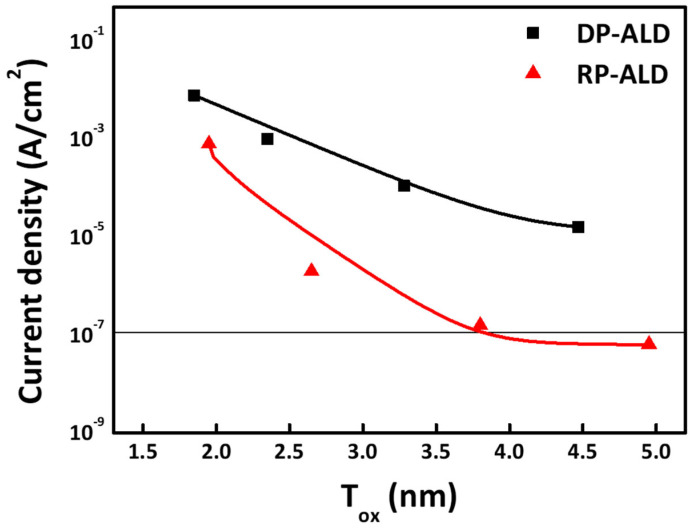
Current densities of HfO_2_ thin films fabricated via DP-ALD and RP-ALD as a function of EOT.

Figure 10 presents the leakage current characteristics of HfO_2_-based capacitors fabricated via DP-ALD and RP-ALD. The plot shows the current density at 0.8 V as a function of equivalent oxide thickness (EOT), categorized by plasma type. For clarity, solid lines are included to guide the eye, and data points are color-coded: black for DP and red for RP. The results demonstrate that, at the same physical thickness, the RP-deposited films exhibit significantly lower leakage currents than their DP counterparts. Specifically, at an EOT of approximately 2.4 nm, the HfO_2_ capacitor fabricated via the RP process exhibits a leakage current density of approximately 1 × 10^−6^ A/cm^2^, whereas the DP-processed counterpart exhibits a considerably higher value of approximately 9 × 10^−4^ A/cm^2^. These findings indicate that the RP-ALD process induces substantially fewer plasma-related defects, resulting in a reduced number of interface and bulk defects. Consequently, superior leakage performance can be achieved even at comparable EOT levels.

## 4. Conclusions

In this work, we investigated and compared the characteristics of HfO_2_ thin films deposited on trench-structured silicon substrates using DP-ALD and RP-ALD. While DP-ALD achieved higher deposition rates, RP-ALD offered superior conformality and film uniformity, achieving nearly 100% step coverage regardless of trench orientation. XPS analysis confirmed the presence of a higher density of non-lattice Hf and oxygen-related defects in DP-ALD films, attributable to direct plasma exposure. These defects resulted in increased leakage current, despite a slight enhancement in capacitance due to the defect–polarization effect. By contrast, RP-ALD significantly suppressed defect formation by eliminating direct plasma contact, thereby significantly improving dielectric reliability and reducing leakage current at equivalent EOT. Overall, although DP-ALD is advantageous for productivity, RP-ALD demonstrates distinct benefits in terms of film quality and electrical performance. These findings highlight the importance of plasma configuration in ALD processes and establish RP-ALD as a more suitable technique for the reliable integration of high-k dielectrics in advanced DRAM capacitor applications. Furthermore, this study is limited to HfO_2_ and a specific trench structure. Future research investigating the applicability of this process to various high-k materials and more complex 3D structures with high aspect ratios (e.g., 100:1), along with evaluations of long-term reliability and in-depth analyses of defect formation mechanisms, could enable the extension of this technique to next-generation plasma-based dielectric processes for DRAM applications.

## Data Availability

Data are contained within the article.

## Abbreviations

The following abbreviations are used in this manuscript:

CD	critical dimension
DRAM	dynamic random-access memory
ALD	atomic layer deposition
MIM	metal–insulator–metal
RP-ALD	remote plasma ALD
DP-ALD	direct plasma ALD
EOT	equivalent oxide thickness
ZAZ	ZrO_2_/Al_2_O_3_/ZrO_2_
